# Supplementary material for: Molecular Dynamics Simulation of Transport Mechanism of Graphene Quantum Dots through Different Cell Membranes
Source: Membranes (Basel). 2022 Jul 31;12(8):753. doi: 10.3390/membranes12080753 (PMC9414618; doi:10.3390/membranes12080753)
Supplement: Supplementary file 1 [file membranes-12-00753-s001.zip › membranes-1767506-SI.pdf]

membranes

Supplementary Materials

Molecular Dynamics Simulation of Transport Mechanism of Graphene Quantum Dots Through Different Cell Membranes

Pengzhen Zhang [1,†], Fangfang Jiao [2,†], Lingxiao Wu [1], Zhe Kong [1,*], Wei Hu [2,*], Lijun Liang [3] and Yongjun Zhang [1]

1. Center of Advanced Optoelectronic Materials and Devices, Key Laboratory of Novel Materials for Sensor of Zhejiang Province, College of Materials and Environmental Engineering, Hangzhou Dianzi University, Hangzhou 310018, China; 13519288162@163.com (P.Z.), wlx_1171999418@163.com (L.W.), yjzhang@hdu.edu.cn (Y.Z.)
2. Shandong Provincial Key Laboratory of Molecular Engineering, School of Chemistry and Pharmaceutical Engineering, Qilu University of Technology (Shandong Academy of Sciences), Jinan 250353, People's Republic of China, jiao1313@outlook.com
3. College of Automation, Hangzhou Dianzi University, Hangzhou 310018, China, michael.lijunl@gmail.com

† P. Z. Z and F. F. J. contributed equally to this work.
* Correspondence: zhekong@hdu.edu.cn (Z.K.); weihu@qlu.edu.cn (W.H.) Fax: +86 571 87713538

Table S1. Details of simulation calculations.

System	No. of atoms	No. of water molecules	Simulation method	GQD:lipid	Time (ns)
GQDs-POPC	117,653	26,939	MD	1:274	200
GQDs-DOPE	126,576	29,624	MD	1:316	200
GQDs-POPE	129,756	27,902	MD	1:342	200
GQDs-POPE-LD	138669	31933	MD	1:342	200
GQDs-POPC	117,653	26,939	SMD	1:274	10
GQDs-DOPE	126,576	29,624	SMD	1:316	10
GQDs-POPE	129,756	27,902	SMD	1:342	10
GQDs-POPE-LD	138669	31933	SMD	1:342	10
GQDs-POPC	117,653	26,939	PMF	1:274	S*10
GQDs-DOPE	126,576	29,624	PMF	1:316	S*10
GQDs-POPE	129,756	27,902	PMF	1:342	S*10
GQDs-POPE-LD	138669	31933	PMF	1:342	S*10

MD Simulation

MD simulations were performed using Gromacs5.0 software [1]. The charge of the carbon atom at the edge of GQDs was set to +1.15e; the charge of the hydrogen atom connected to the carbon atom was set to -1.15e, and the remaining carbon atom charge was considered neutral [2]. The relevant parameters of GQDs, including the harmonic bond potentials of C-C and C-H, the harmonic angles of C-C-H and C-C-C, the harmonic dihedral potential, and Lennard-Jones (LJ), were taken from ref [3]. LJ and charge parameters are listed in Table 2.These parameters have been successfully used to explore the interaction between GQDs and DNA, and the transmembrane transport of GQDs [2,4–7]. All systems were subjected to 50,000 steps of energy minimization and 1 ns pre-equilibration before the MD simulation. All bond lengths and all angles involving hydrogen atoms were constrained using the LINCS algorithm. The NPT ensemble was used in the simulation with a constant temperature of 314 K imposed by a Berensdsen thermostat and 1 bar of pressure controlled by a semi-isotropic Parrinello–Rahman barostat. The cutoff for the nonbonded van der Waals interaction was set by a switching function starting at 1.0 nm and reaching zero at 1.2 nm. The long-range electrostatic interaction was calculated by particle mesh Ewald (PME) summation, with a cutoff of 1.2 nm for the separation of the direct and reciprocal space summation.

Table S2. The LJ and charge parameters used in the MD

Element	C(sp2)	C_{CH}	H_{CH}
ε (kcal/mol)	0.0859	0.046	0.0301
σ (Å)	3.3997	2.985	2.42
q(e)	0	−0.115	0.115
Reference	[8]	[9]	[9]

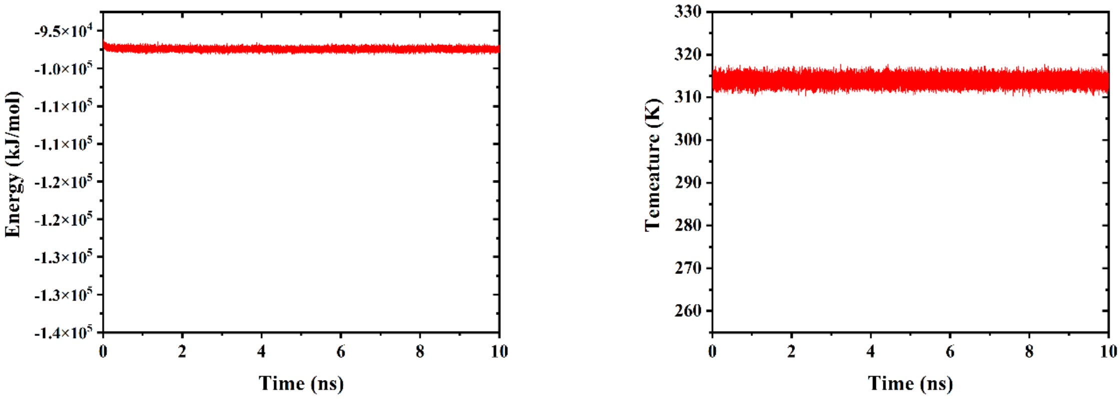

Figure S1. Changes in energy and temperature of the system.

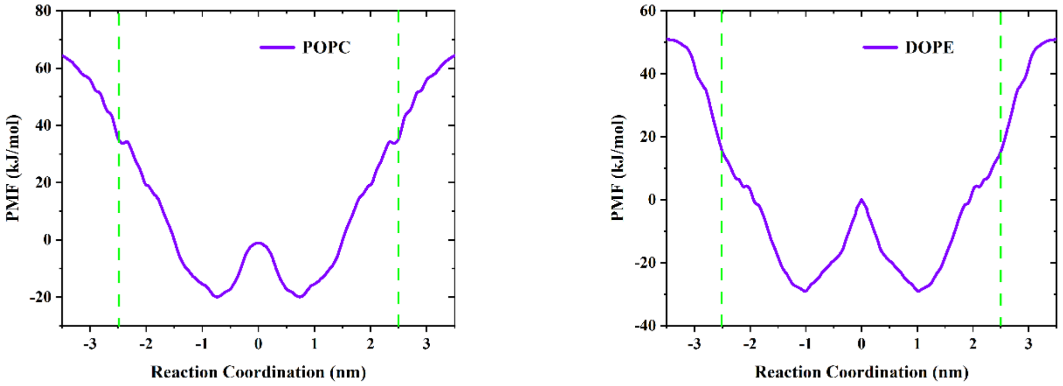

Figure S2. The average force (PMF) potential of GQDs transport through the membrane. The green dotted line indicates the two boundaries of the cell membrane.

References

1. Mja, A.; Tm, D.; Rsb, C.; Sp, A.; Jcsb, C.; Bh, A.; Ela, D. GROMACS: High performance molecular simulations through multi-level parallelism from laptops to supercomputers - ScienceDirect. *SoftwareX* **2015**, *1–2*, 19–25, doi:10.1016/j.softx.2015.06.001.
2. Liang, L.; Kong, Z.; Kang, Z.; Wang, H.; Zhang, L.; Shen, J.W. Theoretical Evaluation on Potential Cytotoxicity of Graphene Quantum Dots. *ACS Biomater. Sci. Eng.* **2016**, *2*, 1983–1991, doi:10.1021/acsbiomaterials.6b00390.
3. Cohen-Tanugi, D.; Grossman, J.C. Water Desalination across Nanoporous Graphene. *Nano Lett.* **2012**, *12*, 3602–3608, doi:10.1021/nl3012853.
4. Kong, Z.; Hu, W.; Jiao, F.; Zhang, P.; Shen, J.; Cui, B.; Wang, H.; Liang, L. Theoretical Evaluation of DNA Genotoxicity of Graphene Quantum Dots: A Combination of Density Functional Theory and Molecular Dynamics Simulations. *J. Phys. Chem. B* **2020**, *124*, 9335–9342, doi:10.1021/acs.jpcb.0c05882.
5. Li, J.; Zhang, Y.; Yang, J.; Bi, K.; Ni, Z.; Li, D.; Chen, Y. Molecular dynamics study of DNA translocation through graphene nanopores. *Phys Rev E Stat Nonlin Soft Matter Phys* **2013**, *87*, 062707, doi:10.1103/PhysRevE.87.062707.

6. Zhang, L.; Xu, B.; Wang, X. Cholesterol Extraction from Cell Membrane by Graphene Nanosheets: A Computational Study. *J. Phys. Chem. B* **2016**, *120*, 957–964, doi:10.1021/acs.jpcb.5b10330.
7. Chen, J.; Zhou, G.; Chen, L.; Wang, Y.; Wang, X.; Zeng, S. Interaction of Graphene and its Oxide with Lipid Membrane: A Molecular Dynamics Simulation Study. *J. Phys. Chem. C* **2016**, *120*, 6225–6231, doi:10.1021/acs.jpcc.5b10635.
8. Beu, T.A. Molecular dynamics simulations of ion transport through carbon nanotubes. I. Influence of geometry, ion specificity, and many-body interactions. *J. Chem. Phys.* **2010**, *132*, 164513, doi:10.1063/1.3387972.
9. Mooney, D.A.; Müller-Plathe, F.; Kremer, K.J.C.P.L. Simulation studies for liquid phenol: properties evaluated and tested over a range of temperatures. *Chem. Phys. Lett.* **1998**, *294*, 135–142, doi:10.1016/S0009-2614(98)00860-4.
